# ﻿*Wikstroemiafragrans* (Thymelaeaceae, Daphneae), a new species from Mount Danxia, China based on morphological and molecular evidence

**DOI:** 10.3897/phytokeys.213.91116

**Published:** 2022-11-10

**Authors:** Jing-Rui Chen, Shiou Yih Lee, Jian-Qiang Guo, Jie-Hao Jin, Qiang Fan, Wen-Bo Liao

**Affiliations:** 1 State Key Laboratory of Biocontrol, School of Life Sciences, Sun Yat-Sen University, Guangzhou 510275, China; 2 Guangdong Provincial Key Laboratory of Plant Resources, School of Life Sciences, Sun Yat-Sen University, Guangzhou 510275, China; 3 Faculty of Health and Life Sciences, INTI International University, Nilai 71800, Malaysia; 4 Administrative Commission of Danxiashan National Park, Shaoguan 512300, China

**Keywords:** Danxia landform, flora, internal transcribed spacer (ITS), IUCN Redlist, phylogenetics, taxonomy, *Wikstroemia*

## Abstract

A new species, *Wikstroemiafragrans* (Thymelaeaceae, Daphneae), from Danxiashan National Park, Shaoguan, Guangdong of China is described and illustrated. It is similar to the sympatric *W.trichotoma*, but can be differentiated easily from the latter by its shorter racemose inflorescences, yellowish green calyx tube, and smaller leaves. It also resembles the allopatric *W.fargesii*, but differs from it by its strigose-pubescent ovary and disk scale that is 2- or 3-dentate apically. Phylogenetic analysis using the nuclear DNA internal transcribed spacer (ITS) region revealed that *W.fragrans* falls within the *Wikstroemia* clade; based on current sampling, *W.fragrans* is closely-related to *W.capitata*. It is also the first species of *Wikstroemia* known to be endemic to the Danxia landform and is classified provisionally as Critically Endangered according to the IUCN Red List Categories and Criteria.

## ﻿Introduction

Thymelaeaceae comprise 50 genera and about 900 species widely distributed in both temperate and tropical regions (Herber 2002, 2003). It is circumscribed to include three subfamilies: Thymelaeaoideae (ca. 42 genera), Octolepidoideae (8 genera), and a yet to be validly published “Tepuianthoideae” (1 genus). *Wikstroemia* Engl. is a member of the Daphne group (Daphneae) of the Thymelaeaoideae, comprising approximately 70 species. *Wikstroemia* is widely distributed in Asian and Oceanic regions, with some populations scattered around the Hawaiian Islands. Among these species, 54 were reportedly naturally distributed in China (Rogers 2009-onwards).

Since 2004, we have conducted a series of biological surveys on Mount Danxia to elucidate biodiversity patterns in the Danxia landform. Mount Danxia in Guangdong, China, from which the name Danxia landform is derived, is characterized by steep slopes derived from sandstones and conglomerates (Peng et al. 2018). Owing to its unique geological and geomorphic structure, the ecological succession of biota is strongly differentiated and a variety of ecosystems appear in a small scale. Mount Danxia harbors a handful of endemic plant species, of which more than ten have been discovered in the last decade, including the recently published *Selaginellaorientali-chinensis* Ching & C.F. Zhang ex H.W. Wang & W.B. Liao (Selaginellaceae) (Wang et al. 2022) and *Lespedezadanxiaensis* Q. Fan, W.Y. Zhao & K.W. Jiang (Fabaceae) (Zhao et al. 2021). The discovery of these species serves to reveal the biodiversity richness of Mount Danxia.

During one of our floristic inventories in Danxiashan National Park in 2022, a plant species that most closely resembled *Wikstroemia* was found. It was previously overlooked and misidentified as *W.nutans* Champion ex Benth, which is a common species widely distributed in Guangdong. The 5-lobed calyx obviously differed from the 4-lobed calyx of *W.nutans*. After careful morphological comparison using herbarium specimens, digital images, and relevant literature of other similar species, we confirmed that our specimens represented an undescribed species, which we here describe as *W.fragrans*. Taxonomic information, including the distribution, habitat, phenology, etymology, and the International Union for Conservation of Natures (IUCN) preliminary conservation status also are provided. The epithet, fragrans, refers to the attractive scent of the flowers, which is like a mixed aroma of orchid and jasmine. A molecular phylogenetic analysis based on the nuclear DNA internal transcribed spacer (ITS) was conducted to evaluate the phylogenetic position and relationship of *W.fragrans* within *Wikstroemia*.

## ﻿Materials and methods

### ﻿Morphological study

The morphological characters of *Wikstroemiafragrans* were compared with similar species, using living plants, relevant literature, and herbarium specimens, including the Chinese Virtual Herbarium (https://www.cvh.ac.cn/) and the China Field Herbarium (https://www.cfh.ac.cn/). Morphological examination was conducted in the Herbarium of Sun Yat-Sen University (SYS). Herbarium acronyms are based on those reported by Thiers (2022).

### ﻿Taxon sampling and molecular analyses

Three individuals of *Wikstroemiafragrans* were collected in Danxiashan National Park, Guangdong, China, during the March to April flowering season in 2022. Voucher specimens were deposited in SYS. The ITS region was used for phylogenetic reconstruction of *Wikstroemia*. Despite insufficiency in delimiting plants at the species level, the ITS regions of most *Wikstroemia* species are publicly available and partly provide an insight into phylogenetic relationships between closely related species (He et al. 2021; Lee et al. 2022). We downloaded all species of Daphneae (Thymelaeaceae) with ITS gene sequences available in NCBI GenBank for analysis. In total, 24 taxa were selected, including 17 species of *Wikstroemia*. Two closely-related species, *Aquilariasinensis* (Lour.) Spreng. (Thymelaeaceae, Aquilarieae) and *Edgeworthiachrysantha* Lindl. (Thymelaeaceae, Daphneae) were included as outgroups. The GenBank accession numbers for each species used in this study are listed in Suppl. material 1.

Total genomic DNA was extracted from silica-gel-dried leaves using the modified cetyltrimethyl ammonium bromide (CTAB) protocol (Doyle and Doyle 1987). The quality and quantity of the DNA extract were determined using Nanodrop spectrophotometer (Thermo Fisher, USA). Polymerase chain reaction was carried out based on the program setting as proposed by Lee et al. (2022) using the universal primers, ITS1 and ITS4 (White et al. 1990). The amplicon was verified under UV and the PCR product was Sanger sequenced both forward and reverse directions. The ITS sequence was assembled and multiple sequence alignment was performed using ClustalW which is embedded in MEGA-X (Kumar et al. 2018). The sequences in the alignment were manually trimmed, in which the nucleotides that correspond to the primer regions were removed from the sequences to obtain a clean sequence read. Phylogenetic trees were reconstructed using the maximum likelihood (ML) and Bayesian inference (BI) methods via MEGA-X (Kumar et al. 2018) and MrBayes v3.2.7a (Ronquist et al. 2012), respectively. For ML, the optimum DNA substitution model calculated using the “Find best DNA/Protein Models (ML) function embedded in MEGA-X was Kimura two-parameter model (K2P) with gamma incorporated (+G) (=K2P+G). All branch nodes were calculated with 1000 bootstrap replicates. For BI, a mixed substitution type and a four by four nucleotide substitution model were selected for the likelihood model, and the Markov chain Monte Carlo simulations were run twice independently for 2000000 generations. Four chains were selected and sampling of data was conducted every 100 generations.

## ﻿Results

### ﻿Morphological comparison

*Wikstroemiafragrans* is similar to the sympatric *W.trichotoma* (Thunb.) Makino. The two species share identical features such as papery leaves, a glabrous calyx tube, 5-lobed calyx, and a lobed disk scale. However, *W.fragrans* differs from the latter by its smaller leaves (1.2–1.6×0.5–0.9 vs. 1.2–3.5(-8) × (0.5-)1–2.2(-4) cm), densely racemose to capitate inflorescence (vs. loose panicle), yellowish green calyx (vs. white), and strigose-pubescent ovary (vs. apically strigose ovary) (Table 1).

**Table 1.** Comparison of morphological features and distribution between *Wikstroemiafragrans*, *W.capitata, W.fargesii*, and *W.trichotoma*.

Characters	*W.fragrans*	W.capitata*	W.fargesii*	W.trichotoma*
Leaf shape	ovate to ovate-lanceolate	elliptic or obovate-elliptic, rarely obovate-oblong	elliptic, suborbicular, or oblong-lanceolate	ovate to ovate-lanceolate
Leaf size (cm)	1.2–1.6 × 0.5–0.9	1–2 × 0.4–1.0	1–2.2 × 0.8–2.0	1.2–3.5(-8) × (0.5–)1–2.2(-4.0)
Inflorescences	densely racemose to nearly capitate, 4–8-flowered	capitate, 3–7-flowered	capitate, 7–10-flowered	a panicle of spikes, few to 10(-26)-flowered
Indumentum of tube abaxially	Glabrous	sericeous-strigose	glabrous	glabrous
Number of calyx lobes	Five	four	five	five
Color of calyx	yellowish green	yellowish green	yellowish green	white, rarely yellowish green
Indumentum of ovary	strigose-pubescent	strigose-pubescent	apically strigose	apically strigose
Shape of subgynoecial disk scale	linear or linear-oblong, apex 2- or 3-dentate	linear, apex 2- or 3-dentate	linear, apex entire or slightly retuse	linear or linear-oblong, membranous, lobed or truncate
Distribution (Province)	Guangdong	Guizhou, Hubei, Shanxi, Sichuan	Chongqing, Hunan**	Anhui, Guangdong, Guangxi, Hunan, Jiangxi, Zhejiang

*Characters of *Wikstroemiacapitata, W.fargesii*, and *W.trichotoma* are from the original descriptions in *Flora of China* (2007). **We checked the herbarium specimens of *Wikstroemiafargesii* collected from Mount Tianmen, Hunan (CSFI026054, CSFI034280) and confirmed their identifications.

Among the *Wikstroemia* species known from China, *W.fragrans* also resembles the allopatric and stenochoric *W.fargesii* (Lecomte) Domke: both species have capitate inflorescences, yellowish green calyces, glabrous tubes, 5-lobed calyces, and a linear disk scale. However, *W.fragrans* differs from *W.fargesii* by its strigose-pubescent ovary (vs. apically strigose ovary) and disk scale 2- or 3-dentate apically (vs. entire or slightly retuse apically) (Table 1).

### ﻿Molecular analysis

**Figure 1.**
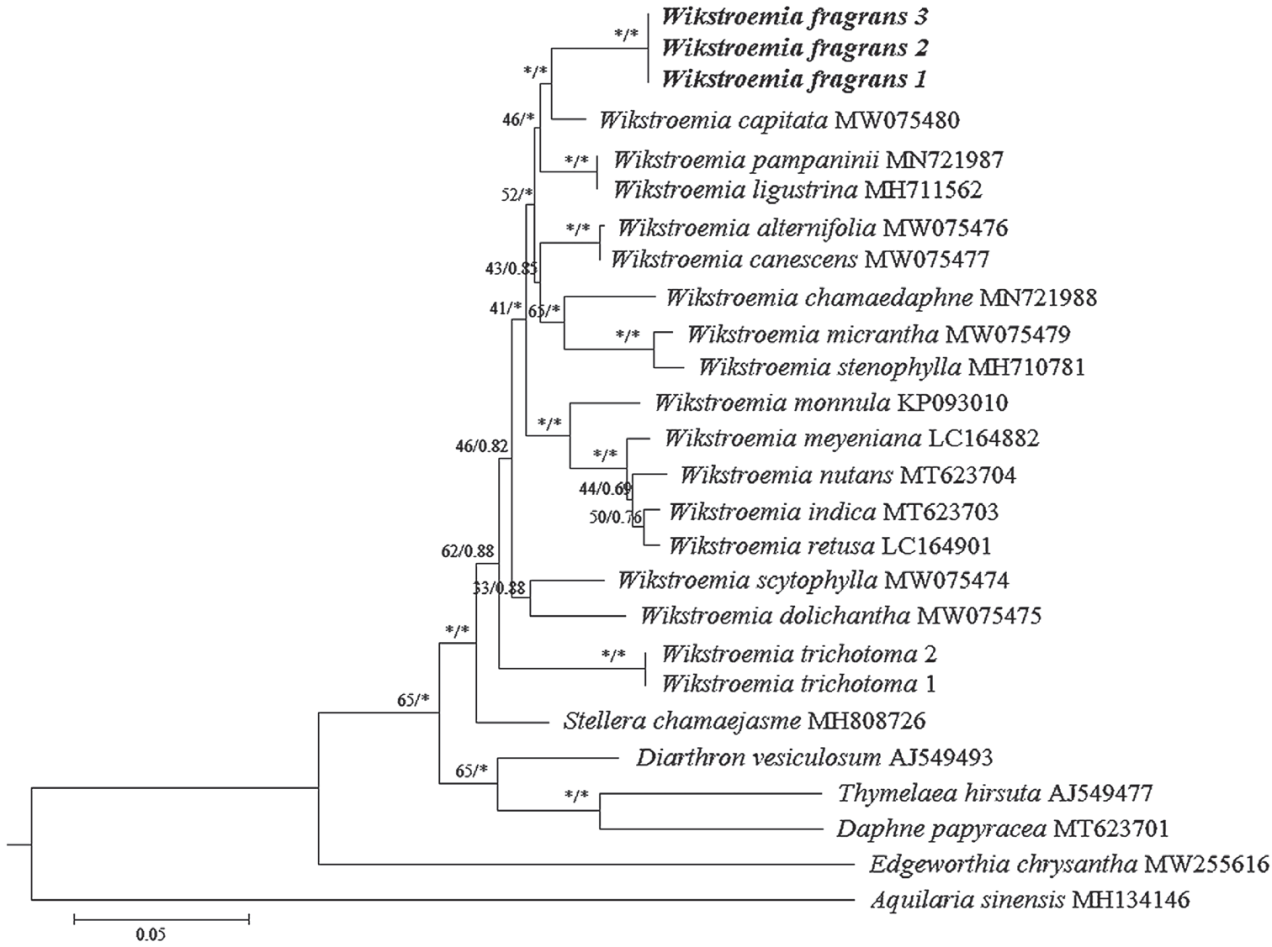
Phylogenetic inference of *Wikstroemiafragrans* and related species based on ITS sequences. Values of Bootstrap support (BS) and posterior probability (PP) are labeled at each branch node, in which BS ≥ 75% and PP ≥ 0.90 are indicated with an asterisk (*). *Wikstroemiafragrans*, described in this study, is shown in bold.

The final sequence alignment based on the ITS dataset was 687 bp. Both the ML and BI trees revealed identical topologies; thus, the trees were merged and only the ML tree is displayed (Fig. 1). The backbone of the phylogenetic tree was not well-supported when using both the ML and BI methods, in which the bootstrap support (BS) value was less than 75% and the posterior probability was less than 0.90. Based on current sampling, species of *Wikstroemia* are monophyletic; *W.fragrans* was placed close to *W.capitata* and the divergence between the two species was strongly supported (BS = 75%, PP = 0.98). *Wikstroemiatrichotoma* was placed distant from *W.fragrans* (Fig. 1).

### ﻿Taxonomic treatment

#### ﻿ Wikstroemia fragrans

Taxon classificationPlantaeMalvalesThymelaeaceae

W.B.Liao, Q.Fan & J.R.Chen sp. nov.

0B084985-6A13-5365-BB65-E1F35B0339B6

urn:lsid:ipni.org:names:77307946-1

Figs 2 , 3 , 4

##### Type.

China. Guangdong Province, Danxia National Park, 25.0°N, 113.7°E, 74 m alt., 16 March 2022 [fl.], *Qiang Fan*, *DNPC 1597* (Holotype SYS! Barcode SYS00236854, Isotypes SYS! Barcode SYS00236855, CSFI!).

##### Diagnosis.

*Wikstroemiafragrans* differs from *W.trichotoma*, by its smaller leaves (1.2–1.6×0.5–0.9 vs. 1.2–3.5(-8) × (0.5-)1–2.2(-4) cm), densely racemose to nearly capitate inflorescence (vs. loose panicle), yellowish green calyx (vs. white), and strigose-pubescent ovary (vs. apically strigose ovary). *Wikstroemiafragrans* differs from *W.fargesii* by its strigose-pubescent ovary (vs. apically strigose) and a disk scale 2- or 3-dentate apically (vs. entire or slightly retuse apically).

##### Description.

Shrub, 0.6–1.6 m tall; perennial branches reddish brown, rugose, annual branches yellowish green, glabrous. ***Leaves*** opposite or subopposite, ovate to ovate-lanceolate, 1.2 – 1.6×0.5 – 0.9 cm, thinly papery, grayish green adaxially, light yellowish green abaxially, glabrous on both surfaces, apex acuminate or obtuse, margin entire, base cuneate or subrounded, midrib flat adaxially, prominent abaxially, secondary veins 4–8 pairs per side, slightly prominent abaxially; petiole ca. 1 mm long. ***Inflorescence*** 4–8-flowered, densely racemose to capitate; peduncle 5 – 15 mm long, glabrous; pedicels absent or ca 0.4–0.7 mm long, glabrous. ***Calyx*** tube yellowish green; 9–11 mm long, exterior glabrous, lobes 5 (sometimes abnormally 6), elliptic, 2.5–3.3×1.2–1.7 mm, margin undulate, glabrous on both surfaces. ***Stamens*** 10 (sometimes abnormally 11), lower whorls of 5 anthers inserted 2–4 mm above middle of hypanthium, upper whorl of 5 anthers at throat; free portion of filaments ca 0.3 mm long; anther linear-oblong, ca 0.8 mm long; subgynoecial disk scale 1, linear or linear-oblong, apex 2- or 3-dentate, 0.8–1.0×0.3–0.7 mm, membranous, glabrous. ***Ovary*** obovoid, 3–4 mm long, ca 0.6 mm in diam., subsessile, strigose pubescent; style ca 0.2 mm long, glabrous; stigma yellow, globose, ca 0.5 mm in diam., surface papillate. ***Drupe*** ca. 6 mm long, yellowish green, ovoid-globose, glabrous, 1-seeded, enclosed by persistent calyx; fruiting pedicel ca 8 mm long. ***Seed*** ovoid, ca. 4 mm long, black, glabrous.

##### Distribution and habitat.

*Wikstroemiafragrans* is currently known only from the type locality, Danxiashan National Nature Reserve (Ba Zhai, Mount Shaoshi, Shuang He Zhai, Yu Nv Lan Jiang), Guangdong, China. It occurs in xerophytic hillside thickets on sandstone and conglomerate based soil at 100–300 m elevation. In this habitat, the most common shrubby and herbaceous species are *Lagerstroemiaindica* Linn. (Lythraceae), *Symplocostanakana* Nakai (Symplocaceae), *Decaspermumgracilentum* (Hance) Merr. et Perry (Myrtaceae), *Violahybanthoides* W. B. Liao & Q. Fan (Violaceae) and *Salviascapiformis* Hance (Lamiaceae).

##### Phenology.

*Wikstroemiafragrans* was observed flowering from March to April, fruiting from April to June.

##### Etymology.

Latin *fragrans*, smell or odor, alluding to sweet-scented flowers. The Chinese name is given as 香花荛花 (xiāng huā ráo huā).

##### Conservation status.

During our intensive floristic inventories in Danxiashan National Nature Reserve from September 2021 to May 2022, only 5 populations of *Wikstroemiafragrans* comprising 5–20 individuals each were found. Due to the limited extent of occurrence (ca. 40 km^2^) and area of occupancy (ca. 5 km^2^) and small population sizes (<100 individuals totally), *W.fragrans* is proposed to be classified as Critically Endangered (CR B1ac(i)+2ac(i)) according to the IUCN Categories (IUCN 2012).

**Figures 2.**
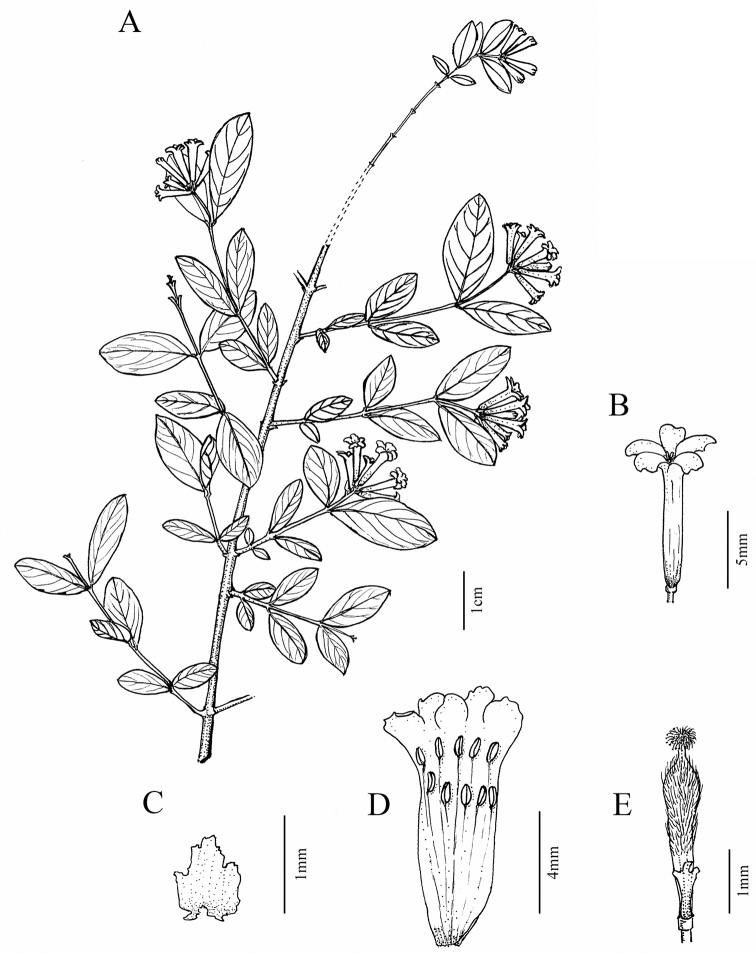
*Wikstroemiafragrans* W.B.Liao, Q.Fan & J.R.Chen, sp. nov. **A** flowering branch **B** flower **C** subgynoecial disk scale opened out **D** dissected flower showing the normal condition of 10 stamens **E** strigose ovary and glabrous disk scale **A–E***DNPC 1597* deposited in SYS. Drawn by Rong-En Wu.

**Figure 3.**
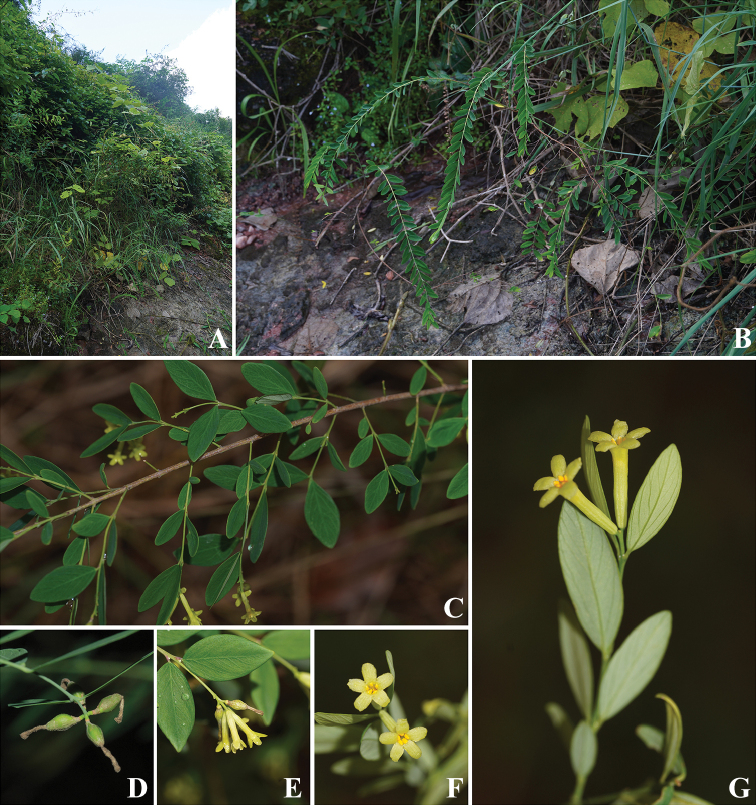
Habitat and morphological features of *Wikstroemiafragrans* W.B.Liao, Q.Fan & J.R.Chen, sp. nov. **A, B** habitat, hillsides thickets of Danxiashan National Park **C** flowering branches **D** immature fruits **E** densely racemose inflorescence **F, G** flowers, showing 5-lobed calyx. (**A–C, E–G** photographs by Jing-Rui Chen of unvouchered plants in the original habitat in April 2022 **D** photograph by Jian-Qiang Guo taken in April 2021).

**Figure 4.**
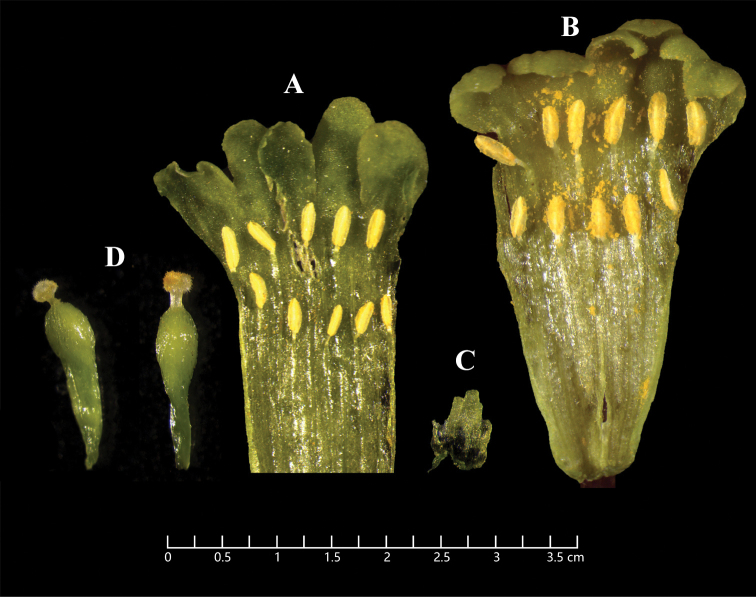
*Wikstroemiafragrans* W.B.Liao, Q.Fan & J.R.Chen, sp. nov. **A** hypanthium dissected showing 10 stamens **B** hypanthium dissected showing abnormal 11 stamens **C** subgynoecial scale disk, linear-oblong, apex 2–3 dentate **D** pistil with strigose-pubescent ovary, glabrous style, and light green stigma. **A–D***DNPC 1597* deposited in SYS.

##### Additional specimens examined (paratypes).

China, Guangdong Province, Danxia National Park, 25.0°N, 113.7°E, 384 m alt., 14 August 2022, *Wan-yi Zhao & Jing-rui Chen*, *DNPC 2966* (SYS); China, Guangdong Province, Danxia National Park, 24.9°N, 113.7°E, 162 m alt., 17 August 2022, *Wan-yi Zhao & Jing-rui Chen*, *DNPC 3029* (SYS).

## ﻿Discussion

The taxonomic status of *Wikstroemia* has long been debated; species exhibit continuous morphological variation, which has complicated efforts to distinguish between species and has created problems in the classification of the genus and its sister genera (Skottsberg 1972; Halda 2001; Wang et al. 2007; Zhang et al. 2007). However, it is generally accepted that *Wikstroemia* can be distinguished from related genera through its inflorescence and scale type (distinct or annular) (Domke 1934; Herber 2002; 2003; Wang et al. 2007).

*Wikstroemiafragrans* was previously confused with *W.nutans*, but can be distinguished easily by the length of its inflorescence axis and number of calyx lobes. After checking all species of *Wikstroemia* distributed in Guangdong, we turned our eyes to the sympatric *W.trichotoma*. These two species share identical features such as papery leaves, a glabrous calyx tube, five calyx lobes, and a lobed disk scale, but differ in leaf size, inflorescence, and calyx color. To support these morphological findings, molecular methods to distinguish closely similar species were also utilized. However, DNA studies of *W.trichotoma* are limited; there is no publicly available record of the ITS sequence of *W.trichotoma*. Thus, we sequenced two individuals of *W.trichotoma* collected from Mount Babao, which is adjacent to Mount Danxia, to be included in our phylogenetic analysis. The phylogenetic tree placed *W.trichotoma* distant from *W.fragrans*, indicating that *W.trichotoma* is at least not considered to be the most immediately genetically affiliated species to *W.fragrans*. Additionally, we noticed that among the Chinese mainland species the allopatric *W.fargesii* is also morphologically similar to *W.fragrans*. We faced enormous difficulties in collecting samples of *W.fargesii*, which had not been collected since the 1890s until it was allegedly rediscovered on limestone in Mount Tianmen, Hunan, but with the help of CSFI, we acquired pieces of flowers and leaves from herbarium specimens of *W.fargesii* from Mount Tianmen. This enabled us to make further comparisons. Dissection of these herbarium materials confirmed the identification of *W.fargesii* and showed differences from *W.fragrans* in the ovary indumentum and shape of the disk scale (Table 1). As the two species grow on different types of substrates (*W.fargesii* on limestone and *W.fragrans* on sandstone and conglomerate from red beds), we are convinced that they represent two independent species.

It is worth mentioning the fragrant flowers of *Wikstroemiafragrans*, which are quite rare in *Wikstroemia*. In general, members of Thymelaeaceae with fragrant flowers are usually found in the sister genus *Daphne*. Based on its fragrance, *W.fragrans* has the potential to be domesticated and cultivated for horticulture purposes. Based on the morphological and molecular evidence obtained through this study, we confirmed that the newly described *W.fragrans* is a distinct species.

## Supplementary Material

XML Treatment for Wikstroemia fragrans

## Supplementary materials

Supplementary material 1 List of the GenBank accession numbers of the ITS sequences of sampled species in this study Data type Phylogenetic. Explanation note The appendix shows the GenBank accession numbers of the ITS sequences of sampled species in this study. https://binary.pensoft.net/file/768258This dataset is made available under the Open Database License (http://opendatacommons.org/licenses/odbl/1.0/). The Open Database License (ODbL) is a license agreement intended to allow users to freely share, modify, and use this Dataset while maintaining this same freedom for others, provided that the original source and author(s) are credited.Jing-Rui Chen, Shiou Yih Lee, Jian-Qiang Guo, Jie-Hao Jin, Qiang Fan, Wen-Bo Liao
