# Supplementary material for: ﻿Wikstroemiafragrans (Thymelaeaceae, Daphneae), a new species from Mount Danxia, China based on morphological and molecular evidence
Source: PhytoKeys. 2022 Nov 10;213:67–78. doi: 10.3897/phytokeys.213.91116 (PMC9836440; doi:10.3897/phytokeys.213.91116)
Supplement: Supplementary material 1 — List of the GenBank accession numbers of the ITS sequences of sampled species in this study [file phytokeys-213-067_article-91116__-s001.docx]

**Appendix 1**. List of the GenBank accession numbers of the ITS sequences of sampled species in this study

| **Species** | **GenBank Accession Number** |
| --- | --- |
| *Aquilaria sinensis* (Lour.) Spreng. | MH134146 |
| *Daphne papyracea* Wall. ex Steud. | MT623701 |
| *Diarthron vesiculosum* C. A. Mey | AJ549493 |
| *Edgeworthia chrysantha* Lindl. | AJ744932 |
| *Stellera chamaejasme* L. | MH808726 |
| *Thymelaea hirsuta* (L.) Endl. | AJ549477 |
| *Wikstroemia alternifolia* Batalin | MW075476 |
| *Wikstroemia canescens* (Wall.) Meisn. | MW075477 |
| *Wikstroemia capitata* Rehd. | MW075480 |
| *Wikstroemia chamaedaphne* Meisn. | MN721988 |
| *Wikstroemia dolichantha* Diels | MW075475 |
| *Wikstroemia fragrans* W. B. Liao & Q. Fan & J. R. Chen | OP021674 OP021675 OP021676 |
| *Wikstroemia indica* (L.) C. A. Mey. | MF063882 |
| *Wikstroemia ligustrina Rehd.* | MH711562 |
| *Wikstroemia meyeniana* Warb. | LC164882 |
| *Wikstroemia micrantha* Hemsl. | MW075479 |
| *Wikstroemia monnula* Hance | KP093009 |
| *Wikstroemia nutans* Champ. ex Benth. | MT623704 |
| *Wikstroemia pampaninii* Rehd. | MN721987 |
| *Wikstroemia retusa* A. Gray | LC164901 |
| *Wikstroemia scytophylla* Diels | MW075474 |
| *Wikstroemia stenophylla* E. Pritz. ex Diels | MH710781 |
| *Wikstroemia trichotoma* (Thunb.) Makino | ON898612 ON898613 |
